# Analytical model of surface second-harmonic generation

**DOI:** 10.1038/s41598-019-39260-9

**Published:** 2019-03-20

**Authors:** Dalibor Javůrek, Jan Peřina

**Affiliations:** Joint Laboratory of Optics of Palacký University and Institute of Physics of the Czech Academy of Sciences, 17. listopadu 12, 771 46 Olomouc, Czech Republic

## Abstract

The process of second-harmonic generation (SHG) in a finite one-dimensional nonlinear medium is analyzed in parallel by the Green-function technique and the Fourier-transform method. Considering the fundamental pump field propagating along a given direction and eliminating back-reflections at the boundaries the terms giving the surface second-harmonic fields in the particular solution of the wave equation are uniquely identified. Using these terms the flow of energy corresponding to the surface second-harmonic fields is analyzed in the vicinity of the boundaries. The formula giving the depth of the nonlinear medium contributing to the surface SHG is obtained. Both approaches for describing the SHG are compared considering complexity and quantization of the interacting fields. In addition, a theoretical model of surface SHG in centrosymmetric media is proposed. The model is built upon assumption that the second-order nonlinearity decays exponentially with distance from the boundary. As an important example, the generation of surface SHG from a thin dielectric nonlinear layer placed on a silicon substrate is analyzed by the proposed model.

## Introduction

The process of second-harmonic generation (SHG) is the first ever observed nonlinear optical process^1^, soon after the discovery of a laser^2^. In its original variant observed by the pioneers of nonlinear optics in^1^, it belongs to bulk second-order nonlinear optical processes observed in general in the volume of non-centrosymmetric nonlinear media. The requirement for non-centrosymmetric nonlinear media arises from the fact that, in electric-field dipole approximation, the symmetry of centrosymmetric nonlinear media prevents the observation of volume SHG.

It has been shown later, that SHG can be observed at an interface between two centrosymmetric media with different material properties^3–6^. The reason is that the symmetry is broken in the vicinity of this interface^4,7,8^: Atoms of both media that are in the contact at the interface change their electronic properties due to the mutual interaction and create this way local nonlinear polarization. This polarization then generates a local second-harmonic field that further propagates through both neighboring media. As the second-harmonic field is apparently emitted from a thin layer around the interface (volume emission is prohibited by the symmetry), we speak about surface SHG, as opposed to the usual volume SHG. The surface SHG has been found useful in diagnostics of material properties of surfaces^9–11^. Recently, the surface SHG has been investigated in relation to metal-dielectric surfaces of nanoparticles^3,4,6^. The reason is that the surface plasmon-polaritons bounded to metal-dielectric surfaces allow for high enhancement of the second-harmonic and/or the fundamental field. Due to the properties of the metal the fields’ enhancement is considerably greater than in the case of only dielectrics and this opens qualitatively new possibilities both in applied photonics and fundamental material science. According to the recent investigations^4^, the volume SHG is not completely prohibited in centrosymmetric media provided that nonzero magnetic-field dipole or electric-field quadrupole contributions exist in the medium. We note that surface nonlinear effects have also been discussed in connection with the process of spontaneous parametric down-conversion and generation of photon pairs^12,13^.

The surface-like SHG can be found as well in the non-centrosymmetric media^14^. In this case, the second-harmonic radiation is also emitted from a thin volume adjacent to a boundary. In comparison with centrosymmetric media this volume is typically an order of magnitude thicker. When Blombergen and Pershan, the authors of the original paper^14^, analyzed the process of SHG in a homogeneous nonlinear non-centrosymmetric finite medium in the standard, i.e. volume, approach, they found out discontinuities of the tangential components of the electric and magnetic fields at the boundaries. As these discontinuities are not allowed by the Maxwell equations, they removed them and arrived at the surface SHG for the first time^14^. Their results were successfully experimentally verified^15^ and later extended^7,16^.

Despite the fact that this phenomenological approach to surface SHG does not directly incorporate local changes of the electronic structure of atoms at the interface and thus neglects certain part of the surface SHG it has been found very useful. The reason is that the underlying formulas are written only for the optical-field amplitudes and so they can be relatively easily applied to more complex nonlinear optical systems. This is especially important under two different situations. First, when complex nonlinear photonic structures are analyzed. In this case, this simplified approach allows to keep certain control above the structure of the nonlinear contributions without sheer relying on the results of complex numerical calculations that involve the dynamics of induced electric dipoles. The second case occurs when quantum properties of the interacting fields are important. Here, the consideration of quantum fields poses the question how to consistently quantize an optical field in the simplest way compatible with the description of the nonlinear process.

In the framework of this phenomenological approach, two different methods have been developed: The first one is based upon the rigorous (and apparently mathematically consistent) approach that uses the Green-function technique. In the second method, the Fourier transform is applied to the interacting fields in the infinite volume to find a general (‘generic’) solution and the boundary conditions are incorporated subsequently using the field amplitudes’ continuity relations at the boundaries.

The second method has been used much more frequently in the past as it is easier for application due to its clearly defined steps that allow for safe implementation even in more complex nonlinear photonic structures (e.g., using the transfer-matrix approach). Moreover, the interacting optical fields in this method may be conveniently quantized in an infinite volume after their decomposition into orthonormal monochromatic plane waves^17^. In fact, this formulation is close to the original description by Blombergen and Pershan who wrote a particular solution of the wave equation for the second-harmonic field in the form of harmonic plane waves and, alternatively, applied the Fourier transform method without considering the boundaries^7,14,15,18^. They arrived at the surface SHG just after considering the continuity requirements for the electric- and magnetic-field amplitudes at the boundaries. The behavior of optical fields at the boundaries is consistent with the optical-field quantization procedure as long as we invoke the quantization of photon-field flux (not energy, as it is usually done in quantum optics)^19^. For this type of quantization, only ‘continuous’ propagation of photons through both the involved linear and nonlinear media is required.

On the other hand, the first phenomenological method gives us the mathematically rigorous formula for second-harmonic field in terms of the Green functions of the nonlinear wave equation considered together with the boundary conditions. The Green function gives the solution with an impulse source function placed at a varying position inside the nonlinear medium^20,21^. So it allows for a physically-sound interpretation based on identifying individual material nonlinear dipoles at the positions of the impulse source functions, where they serve as physical sources of the emitted second-harmonic field. However, the structure of such solution is much less transparent and also the relation to the quantization scheme based on monochromatic plane waves is not straightforward.

Moreover, the analysis of surface SHG performed up to now assumed different indices of refraction for the media at different sides of a boundary. Thus, the nonlinear effects around the boundary were influenced by linear properties of the interacting fields. In the limiting, but common, case of surface SHG generated in photonic structures with more than one boundary, the effects of surface SHG emission and zig-zag propagation of the SHG field are mixed such that they cannot be separated. Both effects can only be safely separated when the effects of linear propagation are eliminated by considering the special case in which both linear and nonlinear media inside a photonic structure share the same index of refraction. Only in this very specific case, that is analyzed in the paper, the genuine properties of the SHG field emitted around the boundary are revealed. In the paper, we analyze this case from the point of view of both methods and show how the surface SHG is incorporated in both of them. Moreover, by comparing side by side all terms contained in two methods, we demonstrate in detail their close structural similarity that results in identical formulas for the second-harmonic field. In this comparison, we clearly identify both photons emitted in the volume and photons emerging at the surfaces of different media. To address all important physical aspects of the SHG process, it is sufficient to analyze the simplest case of collinear (1D) propagation of both fields. This brings us, among others, detailed understanding of the flow of the Poynting vector of the surface second-harmonic field in the vicinity of the interface. This extends the conclusions drawn both in the original papers^14,15^ and recent literature^7,16^. We show that surface SHG propagates in the vicinity of the boundary always in the direction against the pump fundamental field, even inside the nonlinear medium. The overall second-harmonic field, consisting both from the volume and surface contributions, then propagates also against the pump-field direction in the close vicinity of the boundary and begins to co-propagate with the pump field typically several tens of nm beyond the boundary.

In addition, we address in the paper the problem of description of surface SHG in centrosymmetric nonlinear crystals with zero volume *χ*^(2)^ susceptibility. We show that the considered phenomenological model allows to treat such SHG fields by defining a suitable spatial profile of *χ*^(2)^ susceptibility around the boundary. If this profile decays exponentially from the boundary into the volume of the centrosymmetric medium, simple analytical formulas are obtained. Applying this model, surface SHG from a thin layer placed on a silicon substrate is analyzed as an important practical example.

The paper is structured as follows. We formulate and solve the model of SHG in section Second-harmonic generation treated by the Green-function technique first. Then, the model is analyzed in section Second-harmonic generation treated by the Fourier-transform method. The subsequent section brings the Poynting-vector analysis of second-harmonic generation near the boundaries. The model of surface SHG in centrosymmetric media and its application to a nonlinear layer on a substrate is presented in section Surface second-harmonic generation in centrosymmetric crystals. In the last section, Conclusions are drawn.

## Second-Harmonic Generation Treated By The Green-Function Technique

At the phenomenological level, the process of SHG is described by the second-order term in the Taylor expansion of the nonlinear polarization vector **P**^nl^(**r**, *t*) considered as a function of the fundamental (pump) electric-field amplitude vector **E**(**r**, *t*)^7,17,22^, i.e.

1 $${{\bf{P}}}^{{\rm{nl}}}={\varepsilon }_{0}{\chi }^{\mathrm{(2)}}{\bf{E}}{\bf{E}}.$$

In Eq. (1), nonlinear properties of the medium responsible for SHG are described by the second-order nonlinear tensor *χ*^(2)^ and *ε*_0_ means the vacuum permittivity. According to Eq. (1), if the medium is irradiated by a strong fundamental field at frequency *ω*, the induced nonlinear polarization **P**^nl^ generates a second-harmonic field **E** at doubled frequency 2*ω*.

**Figure 1:**
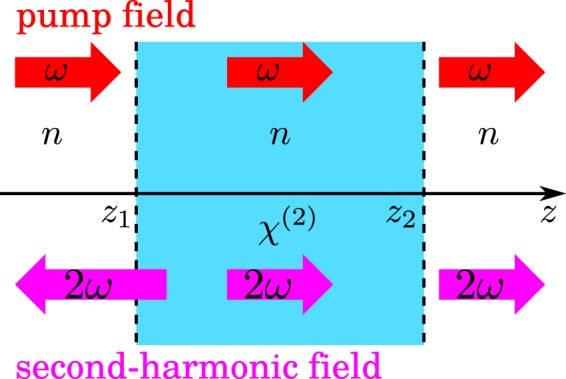
Scheme of the investigated nonlinear structure positioned between *z* = *z*_1_ and *z* = *z*_2_. In our 1D model, the structure is infinite in the transverse directions *x* and *y*. All media have the same linear indices of refraction *n*. The pump beam (with angular frequency *ω*) responsible for SHG impinges on the structure from the left and it propagates only along the +*z* axis (as indicated by the red arrows). Contrary to this, the second-harmonic field (with angular frequency 2*ω*) generated by the nonlinear process propagates both along the −*z* and +*z* axis depending on the position (see the purple arrows). The fact that the second-harmonic field propagates along the −*z* axis not only in the area *z* < *z*_1_ but also for certain *z* > *z*_1_ shows that the surface SHG is not a ‘point process’, but it occurs in certain surroundings of the boundary.

To present the analysis in its simplest form, that, however, keeps all the substantial features of the nonlinear process, we concentrate on 1D propagation of the fundamental and second-harmonic fields (along the *z* axis). We put the nonlinear medium in between the boundaries positioned at *z*_1_ and *z*_2_ (see Fig. 1). We assume all the media being homogeneous and isotropic. Also we consider the fundamental field to be undepleted by the nonlinear process.

Under these conditions, the cartesian components *E*_*j*_, *j* ∈ {x, y, z}, of the second-harmonic field at frequency 2*ω* obey the following form of the wave equation:

2 $$\begin{array}{rcl}\frac{{d}^{2}{E}_{j}(z,\,2\omega )}{d{z}^{2}}-{\delta }_{zj}\frac{{d}^{2}{E}_{j}(z,\,2\omega )}{d{z}^{2}}+\frac{4{\omega }^{2}{n}^{2}\mathrm{(2}\omega )}{{c}^{2}}{E}_{j}(z,\,2\omega ) & = & -\,4\,{\mu }_{0}{\omega }^{2}{P}_{j}^{{\rm{nl}}}(z,\,2\omega ),\\  &  & j\in \{x,\,y,\,z\mathrm{\}.}\end{array}$$

In Eq. (2), *n*(2*ω*) stands for the index of refraction of the second-harmonic field in the nonlinear medium, *c* is the vacuum speed of light, *μ*_0_ means the vacuum permeability, and *δ* denotes the Kronecker symbol. According to Eq. (1) a *j*-th component of the nonlinear-polarization source term ${P}_{j}^{{\rm{nl}}}$ in Eq. (2) is expressed as

3 $${P}_{j}^{{\rm{nl}}}(z,\,2\omega )={\varepsilon }_{0}\sum _{k,l=x,y,z}\,{\chi }_{jkl}^{\mathrm{(2)}}{E}_{k}({\bf{r}},\,\omega ){E}_{l}({\bf{r}},\,\omega )$$

using the fundamental electric-field amplitude **E**(**r**, *ω*).

The solution of the wave equation (2) can be written as a sum of a homogeneous solution **E**^H^(*z*, 2*ω*) and a particular solution **E**^P^(*z*, 2*ω*). In our model, the electric-field amplitude **E**^H^(*z*, 2*ω*) of the homogeneous solution has to be perpendicular to the *z* axis and so it can be decomposed into the plane waves polarized along the *x* and *y* axes propagating in both forward (index F) and backward (B) directions. So, we can express the homogeneous solution **E**^H^ in the form

4 $${{\bf{E}}}^{{\rm{H}}}(z,\,2\omega )=\sum _{a={\rm{F}},{\rm{B}}}\,\sum _{j=x,y}\,{A}_{j}^{a}\mathrm{(2}\omega ){{\bf{e}}}_{j}\,\exp [i{k}^{a}\mathrm{(2}\omega )z\mathrm{].}$$

The complex amplitudes ${A}_{j}^{a}$ belong to plane waves with wave vectors *k*^*a*^ propagating in either forward (*a* = *F*, *k*^*F*^ ≡ *k*) or backward direction (*a* = *B*, *k*^*B*^ ≡ −*k*) and polarized along either the *x* (**e**_*x*_) or *y* (**e**_*y*_) axis. We note that *k*(*ω*) = *n*(*ω*)*ω*/*c*. The complex amplitudes ${A}_{j}^{a}$ are commonly used to describe the fields that enter into the analyzed nonlinear medium through outside at the boundaries at *z*_1_ and *z*_2_ or are scattered by the boundaries^21^.

On the other hand, the particular solution **E**^P^ describes the fields emitted by the sources inside the nonlinear medium, in the process of SHG. The determination of the second-harmonic field thus means to find an appropriate particular solution **E**^P^ (in parallel with the homogeneous one **E**^H^). In this section, we reveal the looked-for particular solution **E**^P^ by the Green-function technique. This technique is based on the determination of the Green function *G*_*j*_(*z*, *z*′, 2*ω*) that satisfies the following equation:

5 $$\begin{array}{ccc}\frac{{d}^{2}{G}_{j}(z,\,z{\rm{^{\prime} }},\,2\omega )}{d{z}^{2}}-{\delta }_{zj}\frac{{d}^{2}{G}_{j}(z,\,z{\rm{^{\prime} }},\,2\omega )}{d{z}^{2}}+\frac{4{\omega }^{2}{n}^{2}(2\omega )}{{c}^{2}}{G}_{j}(z,z{\rm{^{\prime} }},\,2\omega ) & = & \delta (z-z{\rm{^{\prime} }}),\\  &  & j\in \{x,\,y,\,z\};\end{array}$$

symbol *δ* stands for the Dirac function. We note that the Green function *G* is in general a second-order tensor for vectorial tasks. However, in our configuration the only nonzero terms are the diagonal ones. Applying the Green function *G*_*j*_(*z*, *z*′, 2*ω*), we may express a *j*-th electric-field component ${E}_{j}^{{\rm{P}}}$ of the particular solution along the formula

6 $${E}_{j}^{{\rm{P}}}(z,\,2\omega )=-\,{\mu }_{0}4{\omega }^{2}{\int }_{{z}_{1}}^{{z}_{2}}\,dz^{\prime} \,{G}_{j}(z,\,z^{\prime} ,\,2\omega ){P}_{j}^{{\rm{nl}}}(z^{\prime} ,\,2\omega ),\,j\in \{x,\,y,\,z\mathrm{\}.}$$

In Eq. (6) the Green-function components *G*_*j*_ determine the second-harmonic electric field vector **E**^P^(*z*, 2*ω*) at coordinate *z* emitted by nonlinear polarization vector **P**^nl^(*z*′, 2*ω*) at position *z*′. Physically, the Green function relates the second-harmonic field at position *z* with its source represented by a single oscillating nonlinear dipole at position *z*′. Since there is a continuum of dipoles in between the coordinates *z*_1_ and *z*_2_, the overall electric field **E**^P^(*z*, 2*ω*) is given by an integral that adds contributions from all dipoles oscillating in the nonlinear medium.

Explicitly, the *z* component *G*_*z*_(*z*, *z*′, 2*ω*) of the Green function is equal to

7 $${G}_{z}(z,\,z^{\prime} ,\,2\omega )=\frac{{c}^{2}}{{\mathrm{(2}\omega )}^{2}{n}^{2}\mathrm{(2}\omega )}\delta (z-z^{\prime} );\,z^{\prime} \in \langle {z}_{1},\,{z}_{2}\rangle \mathrm{.}$$

It follows from Eqs (6) and (7) that the *z* component (longitudinal) ${E}_{z}^{{\rm{P}}}$ of the second-harmonic electric-field amplitude is nonzero only in the nonlinear medium *z* ∈ (*z*_1_, *z*_2_) where it attains the simple form:

8 $${E}_{z}^{{\rm{P}}}(z,\,2\omega )=-\,\frac{1}{{\varepsilon }_{0}{n}^{2}\mathrm{(2}\omega )}{P}_{z}^{{\rm{nl}}}(z,\,2\omega \mathrm{).}$$

On the other hand, the *x* and *y* components *G*_*x*_(*z*, *z*′, 2*ω*) and *G*_*y*_(*z*, *z*′, 2*ω*) of the Green function are derived in the form of fields propagating to position *z* along the +*z* or −*z* direction from a dipole at position *z*′:

9 $$\begin{array}{ccc}{G}_{j}(z,\,z{\rm{^{\prime} }},\,2\omega ) & = & -\frac{ic}{4\,\omega n(2\omega )}\{\theta (-z+z{\rm{^{\prime} }})\exp [-ik(2\omega )(z-z{\rm{^{\prime} }})]\\  &  & +\theta (z-z{\rm{^{\prime} }})\,\exp [ik(2\omega )(z-z{\rm{^{\prime} }})]\},\,j\in \{x,\,y\};\end{array}$$

*θ*(*z*) stands for the Heaviside step function, i.e. *θ*(*z*) = 1 for *z* > 0, *θ*(*z* = 0) = 1/2 and is zero otherwise.

To obtain the general solution described by the Green functions (7) and (9) explicitly, we assume the fundamental pump field to be composed of monochromatic plane waves with wave vectors *k*^*b*^(*ω*) and complex amplitudes ${A}_{j}^{b}(\omega )$:

10 $${\bf{E}}(z,\,\omega )=\sum _{b={\rm{F}},{\rm{B}}}\,\sum _{j=x,y}\,{A}_{j}^{b}(\omega ){{\bf{e}}}_{j}\,\exp [i{k}^{b}(\omega )z].$$

Then, the particular solution of second-harmonic electric-field amplitude **E**^P^(*z*, 2*ω*) provided by the Green-function technique is written as:

11 $$\begin{array}{rcl}{E}_{j}^{{\rm{P}}}(z,\,2\omega ) & = & \sum _{b,g={\rm{F}},{\rm{B}}}\,\sum _{k,l=x,y}\,\frac{2\omega {d}_{jkl}^{bg}\mathrm{(2}\omega )}{2cn\mathrm{(2}\omega )}\\  &  & \times \{{{\rm{rect}}}_{[-\infty ,{z}_{1}^{-}]}(z)\frac{\exp [\,-\,ik\mathrm{(2}\omega )z]}{\Delta {k}_{+}^{bg}}(\exp [i{\rm{\Delta }}{k}_{+}^{bg}{z}_{2}]-\exp [i{\rm{\Delta }}{k}_{+}^{bg}{z}_{1}])\\  &  & +\,{{\rm{rect}}}_{[{z}_{1},{z}_{2}]}(z)[\frac{\exp [ik\mathrm{(2}\omega )z]}{{\rm{\Delta }}{k}_{-}^{bg}}(\exp [i{\rm{\Delta }}{k}_{-}^{bg}z]-\exp [i{\rm{\Delta }}{k}_{-}^{bg}{z}_{1}])\\  &  & -\,\frac{\exp [\,-\,ik\mathrm{(2}\omega )z]}{{\rm{\Delta }}{k}_{+}^{bg}}(\exp [i{\rm{\Delta }}{k}_{+}^{bg}z]-\exp [i{\rm{\Delta }}{k}_{+}^{bg}{z}_{2}])]\\  &  & +\,{{\rm{rect}}}_{[{z}_{2}^{+},\infty ]}(z)\frac{\exp [ik\mathrm{(2}\omega )z]}{{\rm{\Delta }}{k}_{-}^{bg}}(\exp [i{\rm{\Delta }}{k}_{-}^{bg}{z}_{2}]-\exp [i{\rm{\Delta }}{k}_{-}^{bg}{z}_{1}])\},\,j\in \{x,\,y\};\end{array}$$

12 $${E}_{z}^{{\rm{P}}}(z,\,2\omega )=-\,\sum _{b,g={\rm{F}},{\rm{B}}}\,\sum _{k,l=x,y}\,\frac{{d}_{zkl}^{bg}\mathrm{(2}\omega )}{{n}^{2}\mathrm{(2}\omega )}{{\rm{rect}}}_{[{z}_{1},{z}_{2}]}(z)\exp [i({k}^{b}(\omega )+{k}^{g}(\omega ))z\mathrm{].}$$

In Eqs (11) and (12), ${\rm{\Delta }}{k}_{\pm }^{bg}=\pm k\mathrm{(2}\omega )+{k}^{b}(\omega )+{k}^{g}(\omega )$, ${d}_{jkl}^{bg}\mathrm{(2}\omega )={[{{\boldsymbol{\chi }}}^{\mathrm{(2)}}:{{\bf{e}}}_{k}{{\bf{e}}}_{l}]}_{j}{A}_{k}^{b}(\omega ){A}_{l}^{g}(\omega )$ and symbol: shorthands the susceptibility tensor ***χ***^(2)^ with respect to its last two indices. Function rect_[*a*,*b*]_(*z*) equals 1 for *z* ∈ 〈*a*, *b*〉 and is zero otherwise.

According to Eq. (12), the *z* component ${E}_{z}^{{\rm{P}}}(z,\,2\omega )$ of the second-harmonic electric-field amplitude is nonzero only inside the nonlinear medium where it does not depend explicitly on the positions *z*_1_ and *z*_2_ of the boundaries. Similarly, this property is inherited to the first terms on the third and fourth lines of Eq. (11) that give the *x* and *y* components ${E}_{x}^{{\rm{P}}}(z,\,2\omega )$ and ${E}_{y}^{{\rm{P}}}(z,\,2\omega )$, respectively. Local values of the electric-field amplitudes described by these terms thus do not depend on the positions of the boundaries and so we call these terms as generic, i.e. being general to any geometry of the nonlinear medium. Moreover, these terms contain only the doubled wave vectors *k*(*ω*) of the fundamental field.

Contrary to this, the remaining terms in Eq. (11) involve spatial harmonic variations with wave vectors ±*k*(2*ω*) of the second-harmonic field and explicit dependence on the positions *z*_1_ and *z*_2_ of the boundaries. These terms thus explicitly depend on the geometry of the nonlinear medium. In detail, the terms on the second [fifth] line of Eq. (11) describe the field in front of [beyond] the nonlinear medium as waves with wave vector −*k*(2*ω*) [+*k*(2*ω*)] that originate at the positions *z*_1_ and *z*_2_ of the boundaries. Similarly, the second term on the third [fourth] line of Eq. (11) describes the field inside the nonlinear medium as a harmonic wave with wave vector +*k*(2*ω*) [−*k*(2*ω*)] that originates at the boundary at position *z*_1_ [*z*_2_]. Moreover, two terms in line three [four] that belong to the wave propagating with wave vector +*k*(2*ω*) [−*k*(2*ω*)] sum to zero when evaluated at *z*_1_ [*z*_2_]. All these terms are thus specific, i.e. they have to be derived for the chosen geometry.

This division of all terms into the generic and specific ones reveals the structure of the particular solution for the second-harmonic field provided by the Green-function technique. The knowledge of this structure qualitatively helps us in the analysis of more complex nonlinear structures as well as structures emitting weak, i.e. quantum, fields. It allows to reach the appropriate particular solution in two subsequent steps. In the first step, the generic terms are obtained depending on the type of nonlinear interaction and form of the pump field. In the second step, specific terms are added to the generic terms such that the second ones accord with the boundary conditions. This means that the continuity of optical fields at the boundaries has to be guaranteed. This is typically accomplished by applying the transfer-matrix method. The discussed division is also important for quantum fields. It allows to justify the quantization scheme developed for infinite volume^23^. In this scheme, the quantization is based on the generic terms. If we invoke the photon flux quantization^19^, we immediately get the quantization that is consistent with the propagation of light at the boundary.

## Second-Harmonic Generation Treated by the Fourier-Transform Method

We show in this section, that the Fourier-transform method that first solves the wave equation for a nonlinear medium in the whole infinite space and then incorporates the boundary conditions exactly fits into the structure of the particular solution of the second-harmonic field as described in the last paragraph of the previous section.

In the Fourier-transform method the second-harmonic field **E**^P^(*z*, 2*ω*) is decomposed into harmonic plane waves whose complex amplitudes determine the spatial spectrum ${\tilde{E}}_{j}^{{\rm{P}}}(k,\,2\omega )$, i.e.:

13 $${E}_{j}^{{\rm{P}}}(z,\,2\omega )={\int }_{-{\rm{\infty }}}^{+{\rm{\infty }}}\,dk\,{\mathop{E}\limits^{ \sim }}_{j}^{{\rm{P}}}(k,\,2\omega )\,\exp (ikz).$$

The solution of the wave equation (2) in its Fourier domain is then simply written as:

14 $${\tilde{E}}_{j}^{{\rm{P}}}(k,\,2\omega )=\frac{-{\mu }_{0}{c}^{2}{\mathrm{(2}\omega )}^{2}{P}_{j}^{{\rm{nl}}}(k,\,2\omega )}{-{k}^{2}{c}^{2}+{\delta }_{zj}{k}^{2}{c}^{2}+{\mathrm{(2}\omega )}^{2}{n}^{2}\mathrm{(2}\omega )}\mathrm{.}$$

Considering the fundamental field **E**(*z*, *ω*) in the form (10) and formula (3) for the nonlinear polarization **P**^nl^, the inverse Fourier transform applied to the spectral solution in Eq. (14) gives us the *z* component *E*_*z*_(*z*, 2*ω*) of the second-harmonic field in the form (12) reached by the Green-function method. Contrary to this, the *x* and *y* components of the second-harmonic field are obtained in the following form:

15 $$\begin{array}{ccc}{E}_{j}^{{\rm{P}}}(z,\,2\omega ) & = & {\sum }_{b,g={\rm{F}},{\rm{B}}}\,{\sum }_{k,l=x,y}\,\frac{2\omega {d}_{jkl}^{bg}(2\omega )}{2cn(2\omega )}\exp (i[{k}^{b}(\omega )+{k}^{g}(\omega )]z)\,[\frac{1}{{\rm{\Delta }}{k}_{-}^{bg}}-\frac{1}{{\rm{\Delta }}{k}_{+}^{bg}}],\,j\in \{x,\,y\}.\end{array}$$

The terms written in Eq. (15) coincide with those in Eq. (11) that were identified as the generic terms. Thus, the Fourier-transform method provided us all generic terms of the Green-function approach, which is needed to accomplish the first step in the determination of the particular solution as outlined in the previous section. We note that, from the mathematical point of view, the solution in Eq. (15) also represents a particular solution to the wave equation (2), which is however different from that found by the Green-function technique. We note that this solution was found for the first time by Blombergen and Pershan^14^.

We also remind the striking feature of the solution in Eq. (15): It does not contain the terms evolving with the second-harmonic wave vectors ±*k*(2*ω*). It contains only the terms with doubled spatial frequency *k*(*ω*) of the pump field. Thus the free-field propagation of the second-harmonic field from the emitting nonlinear dipoles is not explicitly visible in the solution. The reason is that, for an infinitely extended nonlinear medium, the second-harmonic waves propagating freely with wave vectors −*k*(2*ω*) from the right-hand side of an arbitrary point *z* completely destructively interfere with those coming from the left-hand side with wave vectors +*k*(2*ω*). As a consequence, the formula in Eq. (15) gives only the electric-field amplitude generated by the local nonlinear polarization **P**^nl^ at position *z*.

The solution in Eq. (15) diverges in the phase-matching limit^14,15^. This divergence is then naturally removed when a finite-length nonlinear medium is analyzed, i.e. when the positions *z*_1_ and *z*_2_ of the boundaries are taken into account. Indeed, the relations

16 $$\mathop{\mathrm{lim}}\limits_{{\rm{\Delta }}k\to 0}\frac{\exp (i{\rm{\Delta }}kz)-\exp (i{\rm{\Delta }}k{z}_{\mathrm{1,2}})}{{\rm{\Delta }}k}=i(z-{z}_{\mathrm{1,2}})$$

when applied to the expressions in the third and fourth lines of the solution (11) reached by the Green-function technique guarantee regularity of the whole formula in Eq. (11).

Now we perform the second step in which we include the boundary conditions at positions *z*_1_ and *z*_2_. This means that we extend the particular solution (15) by a suitable homogeneous solution such that the boundary conditions for the electric and magnetic fields are fulfilled. The looked-for particular solution is such that there are no external second-harmonic fields impinging onto the nonlinear medium from outside. This means that the electric-field components *E*_*j*_ of the looked-for particular solution have the following general form outside the nonlinear medium:

17 $$\begin{array}{lll}{E}_{j}(z,\,2\omega ) & = & {A}_{\mathrm{1,}j}^{{\rm{B}}}\,\exp [\,-\,ik\mathrm{(2}\omega )z],\,z\le {z}_{1},\\ {E}_{j}(z,\,2\omega ) & = & {A}_{\mathrm{3,}j}^{{\rm{F}}}\,\exp [ik\mathrm{(2}\omega )z],\,z\ge {z}_{2},\,j\in \{x,\,y\mathrm{\}.}\end{array}$$

The complex amplitudes ${A}_{\mathrm{1,}j}^{{\rm{B}}}$ and ${A}_{\mathrm{3,}j}^{{\rm{F}}}$ used in Eq. (17), together with the complex amplitudes ${A}_{\mathrm{2,}j}^{{\rm{B}}}$ and ${A}_{\mathrm{2,}j}^{{\rm{F}}}$ occurring in Eq. (18) below, parameterize the general solution in a *j*th area specified in the lower index (1 - in front of the nonlinear medium, 2 - inside the nonlinear medium, 3 - beyond the nonlinear medium). On the other hand, a general solution in the nonlinear medium is written in the form:

18 $$\begin{array}{ccc}{E}_{j}(z,\,2\omega ) & = & {A}_{2,j}^{{\rm{F}}}\,\exp [ik(2\omega )z]+{A}_{2,j}^{{\rm{B}}}\,\exp [\,-\,\,ik(2\omega )z]+\,{E}_{j}^{{\rm{P}}}(z,2\omega ),\\  &  & \,z\in \langle {z}_{1},\,{z}_{2}\rangle ,\,j\in \{x,\,y\}.\end{array}$$

The continuity requirements at the boundaries at *z*_1_ and *z*_2_ leaves us with the following set of four linear algebraic equations for amplitudes ${A}_{\mathrm{1,}j}^{{\rm{B}}}$, ${A}_{\mathrm{2,}j}^{{\rm{F}}}$, ${A}_{\mathrm{2,}j}^{{\rm{B}}}$, and ${A}_{\mathrm{3,}j}^{{\rm{F}}}$:

19 $$(\begin{array}{cccc}{C}_{1}^{\ast } & -\,{C}_{1} & -\,{C}_{1}^{\ast } & 0\\ -\,{C}_{1}^{\ast } & -\,{C}_{1} & {C}_{1}^{\ast } & 0\\ 0 & {C}_{2} & {C}_{2}^{\ast } & -\,{C}_{2}\\ 0 & {C}_{2} & -\,{C}_{2}^{\ast } & -\,{C}_{2}\end{array})(\begin{array}{c}{A}_{1,j}^{{\rm{B}}}\\ {A}_{2,j}^{{\rm{F}}}\\ {A}_{2,j}^{{\rm{B}}}\\ {A}_{3,j}^{{\rm{F}}}\end{array})=(\begin{array}{c}{E}_{j}^{{\rm{P}}}({z}_{1})\\ -\,\frac{i}{k(2\omega )}\frac{{\rm{\partial }}{E}_{j}^{{\rm{P}}}}{{\rm{\partial }}z}({z}_{1})\\ -\,{E}_{j}^{{\rm{P}}}({z}_{2})\\ \frac{i}{k(2\omega )}\frac{{\rm{\partial }}{E}_{j}^{{\rm{P}}}}{{\rm{\partial }}z}({z}_{2})\end{array}),\,{C}_{l}=\exp [ik(2\omega ){z}_{l}],\,l=1,\,2,\,j\in \{x,\,y\}.$$

Applying the Cramer’s rule, we arrive at the following solution:

20 $$\begin{array}{rcl}{A}_{\mathrm{1,}j}^{{\rm{B}}} & = & \sum _{b,g=F,{\rm{B}}}\,\sum _{k,l=x,y}\,\frac{2\omega {d}_{jkl}^{bg}\mathrm{(2}\omega )}{2cn\mathrm{(2}\omega )}\frac{\exp [i{\rm{\Delta }}{k}_{+}^{bg}{z}_{2}])-\exp [i{\rm{\Delta }}{k}_{+}^{bg}{z}_{1}]}{{\rm{\Delta }}{k}_{+}^{bg}},\\ {A}_{\mathrm{2,}j}^{{\rm{F}}} & = & -\,\sum _{b,g=F,{\rm{B}}}\,\sum _{k,l=x,y}\,\frac{2\omega {d}_{jkl}^{bg}\mathrm{(2}\omega )}{2cn\mathrm{(2}\omega )}\frac{\exp [i{\rm{\Delta }}{k}_{-}^{bg}{z}_{1}]}{{\rm{\Delta }}{k}_{-}^{bg}},\\ {A}_{\mathrm{2,}j}^{{\rm{B}}} & = & \sum _{b,g=F,B}\,\sum _{k,l=x,y}\,\frac{2\omega {d}_{jkl}^{bg}\mathrm{(2}\omega )}{2cn\mathrm{(2}\omega )}\frac{\exp [i{\rm{\Delta }}{k}_{+}^{bg}{z}_{2}]}{{\rm{\Delta }}{k}_{+}^{bg}},\\ {A}_{\mathrm{3,}j}^{{\rm{F}}} & = & \sum _{b,g=F,{\rm{B}}}\,\sum _{k,l=x,y}\,\frac{2\omega {d}_{jkl}^{bg}\mathrm{(2}\omega )}{2cn\mathrm{(2}\omega )}\frac{\exp [i{\rm{\Delta }}{k}_{-}^{bg}{z}_{2}])-\exp [i{\rm{\Delta }}{k}_{-}^{bg}{z}_{1}]}{{\rm{\Delta }}{k}_{-}^{bg}},\,j\in \{x,\,y\mathrm{\}.}\end{array}$$

The obtained general solution described by the amplitudes in Eq. (20) coincides with the particular solution ${E}_{j}^{{\rm{P}}}$ obtained by the Green function method in Eq. (11).

At the end, we would like to note that the amplitudes of the homogeneous solution **E**^H^ [see Eq. (20)] that corrects the original particular solution **E**^P^ depend linearly on the nonlinear coupling constant *d*. This means that, from the physical point of view, such homogeneous terms should be automatically considered as a part of the particular solution as they describe the second-harmonic field emerging in the nonlinear process.

When we return back to the particular solution for the second-harmonic field obtained by the Green-function technique and written in Eqs (11) and (12) we may formulate the following physical picture of SHG. The second-harmonic field **E**(*z*, 2*ω*) is given as a coherent superposition of local second-harmonic fields emitted by individual nonlinear dipoles. Since a radiating dipole emits its radiation equally in the forward and backward directions [see the Green function (9) symmetric with respect to the exchange *z* ↔ −*z*] the second-harmonic field is expected in general in both the forward and backward directions. Especially, in our geometry, the second-harmonic field propagating along the −*z* axis from the nonlinear medium (*z* < *z*_1_) is expected. This field is built by the radiation emitted into this direction by the nonlinear dipoles situated near the boundary at *z*_1_. The reason is that such radiation cannot be compensated by the radiation propagating in the opposed direction. However, this argumentation can be applied only to a narrow layer surrounding the boundary. This means that the surface SHG is not a ‘point’ process found just in the plane of the boundary, but it occurs in a narrow layer around the boundary. We note that the original studies by Blombergen *et al*.^14,15^ as well as the newer investigations by Mlejnek *et al*.^16^ did not address this issue.

## Poynting-Vector Analysis of Second-Harmonic Generation Near the Boundaries

In this section, we analyze the formula for the second-harmonic field in Eq. (11) in the vicinity of the boundaries. This allows us to reveal the behavior of the surface contribution to the second-harmonic field. In the investigation, we determine the flow of energy of the second-harmonic field as given by the *z* component *S*_*z*_ of the Poynting vector. We first analyze all terms of the second-harmonic field given in Eq. (11) with respect to the field behavior around the boundary at *z*_1_.

The first term in line two and the second term in line four in Eq. (11) depend on *z*_2_ and so they describe the second-harmonic field propagating from the boundary at position *z*_2_ in the −*z* direction. The second terms in lines two and three contribute to the field around the boundary at position *z*_1_. Physically the most important term is the second one on line two that describes constant flow of energy from the nonlinear medium from the area around *z*_1_ (see Fig. 1) back to the free space (−*z* direction), i.e. in the propagation direction opposed to that of the pump beam. This term quantifies the second-harmonic field arising from the region around the boundary at *z*_1_. Fixing again the indices *jkl* and *bg*, the corresponding constant energy flow component ${S}_{{z}_{1}^{-}}(z)$ is obtained in the form (*z* < *z*_1_):

21 $${S}_{jkl,{z}_{1}^{-}}^{bg}(z,\,2\omega )=-\,\frac{{\varepsilon }_{0}}{4c}\frac{{\mathrm{(2}\omega )}^{2}|{d}_{jkl}^{bg}\mathrm{(2}\omega {)|}^{2}}{n\mathrm{(2}\omega )}\frac{1}{{({\rm{\Delta }}{k}_{+}^{bg})}^{2}}.$$

We note that the energy flow is a scalar quantity in our 1D model. As the energy flow component ${S}_{{z}_{1}^{-}}(z)$ is continuous at the boundary at *z*_1_, it has to flow in the −*z* direction also inside the nonlinear medium, in the close vicinity of *z*_1_. In this area, the energy flow ${S}_{{z}_{1}^{+}}$ is approximately described as:

22 $${S}_{jkl,{z}_{1}^{+}}^{bg}(z,\,2\omega )=-\,\frac{{\varepsilon }_{0}}{2{c}^{2}}\frac{{\mathrm{(2}\omega )}^{3}|{d}_{jkl}^{bg}\mathrm{(2}\omega {)|}^{2}}{{k}^{b}(\omega )+{k}^{g}(\omega )}\frac{1}{{({\rm{\Delta }}{k}_{+}^{bg})}^{2}}\mathrm{Re}\{{[1+i{\rm{\Delta }}{k}_{+}^{bg}(z-{z}_{1})]}^{2}\};$$

Re gives the real part of argument. According to this approximative formula, the expected flow of energy in the +*z* direction occurs only for $z > {z}_{{z}_{1}^{+}}^{bg}$:

23 $${z}_{{z}_{1}^{+}}^{bg}=\frac{1}{{\rm{\Delta }}{k}_{+}^{bg}}.$$

From this position the flow of energy in the +*z* direction naturally increases with the increasing *z* for the phase-matched nonlinear interaction. So, there exists a narrow area close to the boundary at *z*_1_$(z\in \langle {z}_{1},\,{z}_{{z}_{1}^{+}}^{bg}\rangle )$ where the energy flows contra-intuitively in the −*z* direction. This energy belongs to the *surface second-harmonic generation*^14–16^.

To complete the analysis of surface SHG, let us continue to determine the partial Poynting vector ${S}_{{z}_{2}^{-}}(z)$ describing the field propagating back from the area around the boundary at position *z*_2_, as given by the first term in line two and second term in line four in Eq. (11). Its magnitude apparently equals that of the partial Poynting vector ${S}_{{z}_{1}^{-}}(z)$ given in Eq. (21) for the field emitted around the boundary at *z*_1_. Moreover, the surface fields generated around both boundaries at *z*_1_ and *z*_2_ may interfere depending on the length *L* ≡ *z*_2_ − *z*_1_ of the nonlinear medium. This interference then leads to the complete Poynting vector *S* at position *z*_1_ encompassing both surface second-harmonic fields:

24 $$S({z}_{1},\,L)=2{S}_{{z}_{1}^{-}}({z}_{1})[1-\,\cos ({\rm{\Delta }}{k}_{+}^{bg}L)].$$

**Figure 2:**
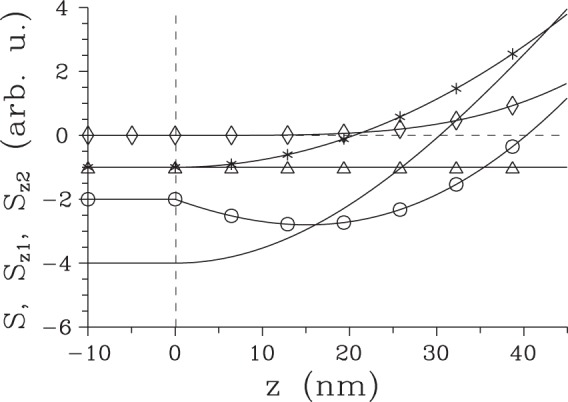
Poynting vector ${S}_{{z}_{1}}$ $[{S}_{{z}_{2}^{-}}]$ of the second-harmonic field originating around the boundary at *z*_1_ = 0 [*z*_2_ ≡ *L*] (solid curve with * [Δ]) and the complete Poynting vector *S* (plain solid curve) as functions of position *z* around the boundary at *z*_1_ = 0 for nonlinear medium of thickness $L=\pi /{\rm{\Delta }}{k}_{jkl}^{bg,(+)}$ giving maximal constructive interference; *n*(*ω*) = 1.5, *n*(2*ω*) = 1.6 and pump central wavelength *λ*(*ω*) = 0.8 *μ*m. For comparison, the complete Poynting vector *S* for *L* = 1 *μ*m [*L* = 0.129 *μ*m] giving partially constructive [completely destructive] interference and attaining minimal values inside the nonlinear material [at the boundary at *z*_1_] is shown (solid curve with ○) [(solid curve with $\diamond$)]. Dashed lines given as *S* = 0 and *z* = 0 are also plotted.

**Figure 3:**
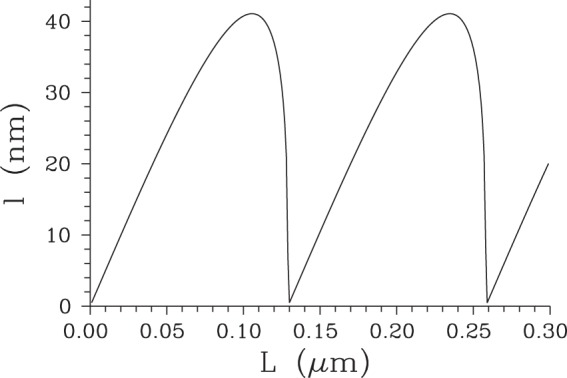
Length *l* of the layer adjacent to the surface at *z*_1_ where the overall surface second-harmonic field flows along the −*z* axis as a function of length *L* of the nonlinear medium; for details, see the caption to Fig. 2.

According to Eq. (24) the overall flow of energy from the nonlinear medium in the −*z* direction is in general nonzero, it vanishes only for specific lengths *L* ($L=2\pi n/{\rm{\Delta }}{k}_{+}^{bg}$, $n\in {\mathbb{N}}$). In this case, the surface second-harmonic field generated around the boundary at ${z}_{1}^{+}$ perfectly compensates that emitted around the boundary at ${z}_{2}^{-}$. Another specific case occurs when both surface contributions maximally constructively interfere [$L=\mathrm{(1}+2n)\pi /{\rm{\Delta }}{k}_{+}^{bg}$, $n\in {\mathbb{N}}$]. The dependence of three analyzed Poynting vectors, ${S}_{{z}_{1}}$, ${S}_{{z}_{2}^{-}}$ and *S*, on position *z* around the boundary at *z*_1_ is plotted in Fig. 2. Assigning sign - to the energy flow in the −*z* direction, the Poynting vector ${S}_{{z}_{1}^{+}}$ only increases with *z*, whereas the Poynting vector ${S}_{{z}_{2}^{-}}$ is constant. The overall Poynting vector *S* attains its minimal value at the boundary at *z*_1_ when the maximally constructive interference between both terms occurs. In this case, the Poynting vector *S* increases with the increasing *z* and it becomes positive at *z* = 32 nm, i.e. at $z > {z}_{{z}_{1}^{+}}$ and ${z}_{{z}_{1}^{+}}$ is defined in Eq. (23). When we change the crystal length *L* such that the constructive interference between two surface second-harmonic fields weakens, the minimum of the overall Poynting vector *S* shifts into the nonlinear medium to position $z\in \langle {z}_{1},{z}_{{z}_{1}^{+}}\rangle$, attains larger values and the area with the negative overall Poynting vector *S* broadens first (see the solid curve with ○ in Fig. 2). Then, this area starts to shrink and the minima of the Poynting vector *S* approach to the value *S* = 0. At certain point, the overall Poynting vector *S* attains only non-negative values. This is the case of the completely destructive interference of two surface second-harmonic fields. If we continue with the change of crystal length *L* further, the negative minimum of the Poynting vector *S* found at the position *z*_1_ sinks down and the extension of the area of *z* with the negative Poynting vector *S* gradually broadens from zero until the case of the completely constructive interference is reached. Gradual evolution of length *l* quantifying the extension of the area with the negative Poynting vector *S* observed for the increasing length *L* of the nonlinear medium in depicted in Fig. 3. According to the curves in Fig. 3, the greatest value of the length *l* equals 42 nm. We note that the discussed interference of surface second-harmonic fields coming from two boundaries can be avoided by either considering non-collinear geometry^15^ or pulsed fields^16^.

## Surface Second-Harmonic Generation in Centrosymmetric Crystals

In centrosymmetric crystals the bulk *χ*^(2)^ susceptibility is zero. As a consequence, the above formulas predict no surface SHG. However, the above presented model can be simply modified to account for the presence of surface SHG in these crystals. At microscopic level, electronic structure of atoms just in the close vicinity of a boundary is modified such that it forms nonlinear dipoles that emit the SHG field^3,4,6^. The more distant the atom from a boundary is, the smaller the modification of its structure. We can phenomenologically describe such behavior by introducing a suitable profile of *χ*^(2)^ susceptibility, e.g. in its simplest exponential form:

25 $${\chi }^{\mathrm{(2)}}(z)={\chi }^{\mathrm{(2)}}({z}_{1})\,\exp (-\,\frac{z-{z}_{1}}{{l}_{{\rm{nl}}}}),\,z\ge {z}_{1},$$

where *l*_nl_ gives the extension of the area with the modified electronic structure of atoms.

Considering this profile in Eqs (3) and (6), we arrive at the modified formulas (11) and (12) for the electric-field amplitudes:

26 $$\begin{array}{rcl}{E}_{j}^{{\rm{P}}}(z,\,2\omega ) & = & {\rm{\exp }}(\frac{{z}_{1}}{{l}_{{\rm{n}}l}})\sum _{b,g={\rm{F}},{\rm{B}}}\,\sum _{k,l=x,y}\,\frac{2\omega {d}_{jkl}^{bg}\mathrm{(2}\omega )}{2cn\mathrm{(2}\omega )}\\  &  & \times \{-\,{{\rm{rect}}}_{[-\infty ,{z}_{1}^{-}]}(z)\frac{{\rm{\exp }}[\,-\,ik\mathrm{(2}\omega )z]}{{\rm{\Delta }}{\tilde{k}}_{+}^{bg}}{\rm{\exp }}[\,i{\rm{\Delta }}{\tilde{k}}_{+}^{bg}{z}_{1}]\\  &  & +\,{{\rm{rect}}}_{[{z}_{1},\infty ]}(z)[\frac{\exp [ik\mathrm{(2}\omega )z]}{{\rm{\Delta }}{\tilde{k}}_{-}^{bg}}({\rm{e}}{\rm{x}}{\rm{p}}[i{\rm{\Delta }}{\tilde{k}}_{-}^{bg}z]-{\rm{e}}{\rm{x}}{\rm{p}}[i{\rm{\Delta }}{\tilde{k}}_{-}^{bg}{z}_{1}])\\  &  & -\frac{\exp [\,-\,ik\mathrm{(2}\omega )z]}{{\rm{\Delta }}{\tilde{k}}_{+}^{bg}}\exp [i{\rm{\Delta }}{\tilde{k}}_{+}^{bg}z]]\},\,j\in \{x,\,y\};\end{array}$$

27 $${E}_{z}^{{\rm{P}}}(z,\,2\omega )=-\,\exp (\frac{{z}_{1}}{{l}_{{\rm{n}}l}})\,\sum _{b,g={\rm{F}},{\rm{B}}}\,\sum _{k,l=x,y}\,\frac{{d}_{zkl}^{bg}\mathrm{(2}\omega )}{{n}^{2}\mathrm{(2}\omega )}{{\rm{rect}}}_{[{z}_{1},\infty ]}(z)\,\exp [i({k}^{b}(\omega )+{k}^{g}(\omega )+i/{l}_{{\rm{nl}}})z];$$

**Figure 4:**
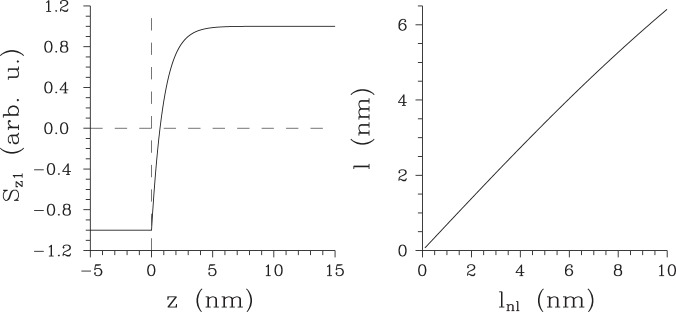
(**a**) Poynting vector ${S}_{{z}_{1}}$ of the second-harmonic field for *χ*^(2)^ profile in Eq. (25) and *l*_nl_ = 1 nm and (**b**) length *l* of the layer with negative Poynting vector ${S}_{{z}_{1}}$ as a function of length *l*_nl_ characterizing the *χ*^(2)^ profile. In (**a**), also dashed lines given as *S* = 0 and *z* = 0 are plotted.

${\rm{\Delta }}{\tilde{k}}_{\pm }^{bg}={\rm{\Delta }}{k}_{\pm }^{bg}+i/{l}_{{\rm{nl}}}$ and the nonlinear coefficient *χ*^(2)^(*z*_1_) at position *z*_1_ is used to determine the nonlinear coefficients ${d}_{jkl}^{bg}$. The results shown in Eqs (26) and (27) were derived for semi-infinite nonlinear medium extending to the right from position *z*_1_. The Poynting vector ${S}_{{z}_{1}}$ drawn for the exponential *χ*^(2)^ profile of Eq. (25) with *l*_nl_ = 1 nm is plotted in Fig. 4(a). We can see in Fig. 4(a) that the length *l* of the layer with the negative Poynting vector ${S}_{{z}_{1}}$ is comparable to the length *l*_nl_. Quantification of this relation is provided in Fig. 4(b) where the curve showing a practically linear dependence of the length *l* of this layer on the length *l*_nl_ is plotted. Comparing qualitatively the curves drawn in Fig. 4 for centrosymmetric crystals on one side and those plotted in Figs. 2 and 3 for non-centrosymmetric crystals on the other side, we observe qualitatively similar behavior of the surface SHG fields in the vicinity of the boundary in both cases. Nevertheless the area of surface SHG in centrosymmetric crystals typically extends over just couple of nm.

Now we consider a nonlinear centrosymmetric crystal positioned in between the coordinates *z*_1_ and *z*_2_. Following the formula in Eq. (25), we describe the *χ*^(2)^(*z*) profile using two exponentials:

28 $${\chi }^{\mathrm{(2)}}(z)={\chi }^{\mathrm{(2)}}({z}_{1})\,\exp (-\,\frac{z-{z}_{1}}{{l}_{{\rm{nl}}\mathrm{,1}}})+{\chi }^{\mathrm{(2)}}({z}_{2})\,\exp (\frac{z-{z}_{2}}{{l}_{{\rm{n}}l\mathrm{,2}}}),\,z\in \langle {z}_{1},\,{z}_{2}\rangle ,$$

and *l*_nl,*j*_ quantifies the extension of *χ*^(2)^ modification around position *z*_*j*_, *j* = 1, 2. Assuming ${l}_{{\rm{nl}}\mathrm{,1}},{l}_{{\rm{nl}}\mathrm{,2}}\ll ({z}_{2}-{z}_{1})$, the corresponding particular solution ${\bar{E}}_{j}^{{\rm{P}}}$ can conveniently be expressed as a superposition of two particular solutions ${E}_{j\mathrm{,1}}^{{\rm{P}}}$ and ${E}_{j\mathrm{,2}}^{{\rm{P}}}$ belonging to the boundaries at *z*_1_ and *z*_2_, respectively:

29 $${\bar{E}}_{j}^{{\rm{P}}}={E}_{j\mathrm{,1}}^{{\rm{P}}}(z,\,2\omega )+{E}_{j\mathrm{,2}}^{{\rm{P}}}(z,\,2\omega ),\,j\in \{x,\,y,\,z\mathrm{\}.}$$

The particular solution ${E}_{j\mathrm{,1}}^{{\rm{P}}}(z,\,2\omega )$ is given by Eqs (26) and (27) in which we substitute *l*_nl_ by *l*_nl,1_. The particular solution ${E}_{j\mathrm{,2}}^{{\rm{P}}}(z,\,2\omega )$ arising from the *χ*^(2)^(*z*) profile around position *z*_2_ is derived in a similar form as that given in Eqs (26) and (27):

30 $$\begin{array}{rcl}{E}_{j\mathrm{,2}}^{{\rm{P}}}(z,\,2\omega ) & = & {\rm{e}}{\rm{x}}{\rm{p}}(-\frac{{z}_{2}}{{l}_{{\rm{nl}}\mathrm{,2}}})\sum _{b,g={\rm{F}},{\rm{B}}}\,\sum _{k,l=x,y}\frac{2\omega {d}_{jkl\mathrm{,2}}^{bg}\mathrm{(2}\omega )}{2cn\mathrm{(2}\omega )}\\  &  & \times \{{{\rm{rect}}}_{[{z}_{2},\infty ]}(z)\frac{\exp [ik\mathrm{(2}\omega )z]}{{\rm{\Delta }}{\tilde{k}}_{-\mathrm{,2}}^{bg}}\exp [i{\rm{\Delta }}{\tilde{k}}_{-\mathrm{,2}}^{bg}{z}_{2}]\\  &  & -\,{{\rm{rect}}}_{[-\infty ,{z}_{2}^{-}]}(z)[\frac{\exp [\,-\,ik\mathrm{(2}\omega )z]}{{\rm{\Delta }}{\tilde{k}}_{+\mathrm{,2}}^{bg}}(\exp [i{\rm{\Delta }}{\tilde{k}}_{+\mathrm{,2}}^{bg}z]-\exp [i{\rm{\Delta }}{\tilde{k}}_{+\mathrm{,2}}^{bg}{z}_{2}])\\  &  & -\,\frac{\exp [ik\mathrm{(2}\omega )z]}{{\rm{\Delta }}{\tilde{k}}_{-\mathrm{,2}}^{bg}}\exp [i{\rm{\Delta }}{\tilde{k}}_{-\mathrm{,2}}^{bg}z]]\},\,j\in \{x,\,y\};\end{array}$$

31 $$\begin{array}{rcl}{E}_{z\mathrm{,2}}^{{\rm{P}}}(z,\,2\omega ) & = & -\,{\rm{e}}{\rm{x}}{\rm{p}}(-\frac{{z}_{2}}{{l}_{{\rm{nl}}\mathrm{,2}}})\sum _{b,g={\rm{F}},{\rm{B}}}\,\sum _{k,l=x,y}\frac{{d}_{zkl\mathrm{,2}}^{bg}\mathrm{(2}\omega )}{{n}^{2}\mathrm{(2}\omega )}{{\rm{rect}}}_{[-\infty ,{z}_{2}]}(z)\\  &  & \times \,\exp [i({k}^{b}(\omega )+{k}^{g}(\omega )-i/{l}_{{\rm{n}}l\mathrm{,2}})z];\end{array}$$

${\rm{\Delta }}{\tilde{k}}_{\pm \mathrm{,2}}^{bg}={\rm{\Delta }}{k}_{\pm }^{bg}-i/{l}_{{\rm{nl}}\mathrm{,2}}$ and the susceptibility *χ*^(2)^(*z*_2_) at position *z*_2_ determines the nonlinear coefficients ${d}_{jkl\mathrm{,2}}^{bg}$.

Up to now, we have strictly assumed identical indices of refraction of all media to eliminate the effects of fields’ back scattering. However, as back-scattering on boundaries separating media with different indices of refraction is an inevitable part of fields’ propagation in real structures, we show here how to generalize the above formulas for a centrosymmetric crystal to account for different indices of refraction in front of and beyond the crystal. We assume that the medium in front of (beyond) the nonlinear crystal [denoted as 1 (3)] has index of refraction *n*_1_ (*n*_3_) whereas the nonlinear crystal [denoted as 2] is characterized by index of refraction *n*_2_. First, we apply the transfer-matrix formalism^21,24^ to the fundamental field to reveal its forward- and backward-propagating components in all three media. This allows us to determine the particular solution ${\bar{E}}_{j}^{{\rm{P}}}$ for the second-harmonic field of the form of Eqs (26) and (27), i.e. without including back-scattering effects. This corresponds to the application of the Fourier-transform method with spatially dependent *χ*^(2)^ nonlinearity. We note that both the forward- and backward-propagating fundamental fields contribute to the solution ${\bar{E}}_{j}^{{\rm{P}}}$. This solution then gives the source terms to the transfer-matrix formulation of the second-harmonic field propagation in the nonlinear crystal. If we assume the *x* polarized second-harmonic electric-field amplitude ${\bar{E}}_{x}^{{\rm{P}}}$, the source terms *S*_1_ and *S*_2_ are given as:

32 $$[\begin{array}{c}{S}_{1}\\ {S}_{2}\end{array}]=-\,{{\mathscr{D}}}_{3}^{-1}\mathrm{(2}\omega ){{\mathscr{D}}}_{2}\mathrm{(2}\omega ){{\mathscr{P}}}_{2}\mathrm{(2}\omega ){{\mathscr{D}}}_{2}^{-1}\mathrm{(2}\omega )[\begin{array}{c}{F}_{1}({z}_{1},\,2\omega )\\ {F}_{2}({z}_{1},\,2\omega )\end{array}]+{{\mathscr{D}}}_{3}^{-1}\mathrm{(2}\omega )[\begin{array}{c}{F}_{1}({z}_{2},\,2\omega )\\ {F}_{2}({z}_{2},\,2\omega )\end{array}]$$

and

33 $${F}_{1}(z,\,2\omega )={\bar{E}}_{x}^{{\rm{P}}}(z,\,2\omega ),\,{F}_{2}(z\mathrm{,2}\omega )=\sqrt{\frac{{\mu }_{0}}{{\varepsilon }_{0}}}{\bar{H}}_{y}^{{\rm{P}}}(z,\,2\omega )=-\,\frac{i}{\omega \sqrt{{\mu }_{0}{\varepsilon }_{0}}}\frac{\partial {\bar{E}}_{x}^{P}(z,\,2\omega )}{\partial z}\mathrm{.}$$

In Eq. (32), the transition matrices ${{\mathscr{D}}}_{l}$ for medium *l*, *l* = 1, 2, 3, and the propagation matrix *P*_2_ for the nonlinear crystal are defined as

34 $${{\mathscr{D}}}_{l}\mathrm{(2}\omega )=[\begin{array}{cc}1 & 1\\ {n}_{l}\mathrm{(2}\omega ) & -\,{n}_{l}\mathrm{(2}\omega )\end{array}],\,{{\mathscr{P}}}_{2}\mathrm{(2}\omega )=[\begin{array}{cc}\exp [i{k}_{2}\mathrm{(2}\omega )(z-{z}_{1})] & 0\\ 0 & \exp [\,-\,i{k}_{2}\mathrm{(2}\omega )(z-{z}_{1})]\end{array}],$$

and the wave vector *k*_2_(2*ω*) belongs to the second-harmonic field inside the crystal.

In the second step, the application of the transfer-matrix formalism provides us the system of two linear algebraic equations for the amplitudes of the reflected ${A}_{\mathrm{1,}x}^{{\rm{B}}}\mathrm{(2}\omega )$ and transmitted ${A}_{\mathrm{3,}x}^{{\rm{F}}}\mathrm{(2}\omega )$ second -harmonic fields at the positions *z*_1_ and *z*_2_, respectively:

35 $$[\begin{array}{c}{A}_{3,x}^{{\rm{F}}}(2\omega )\\ 0\end{array}]={\mathscr{T}}(2\omega )[\begin{array}{c}0\\ {A}_{1,x}^{{\rm{B}}}(2\omega )\end{array}]+[\begin{array}{c}{S}_{1}\\ {S}_{2}\end{array}].$$

In Eq. (35), the source terms *S*_1_ and *S*_2_ are given by Eq. (32) and the transfer matrix ${\mathscr{T}}$ describes linear propagation of the second-harmonic field from the position *z*_1_ to the position *z*_2_ (for details, see^24^). The solution of Eq. (35) is attained in the form

36 $${A}_{1,x}^{{\rm{B}}}(2\omega )=-\frac{{S}_{2}}{{{\mathscr{T}}}_{22}(2\omega )},\,{A}_{3,x}^{{\rm{F}}}=-\frac{{{\mathscr{T}}}_{12}(2\omega )}{{{\mathscr{T}}}_{22}(2\omega )}{S}_{2}+{S}_{1}$$

using the matrix elements ${{\mathscr{T}}}_{jl}$. Finally, the electric-field amplitude *E*_*x*_(*z*, 2*ω*) of the second-harmonic field at an arbitrary position *z* is easily derived from the amplitudes ${A}_{\mathrm{1,}x}^{{\rm{B}}}\mathrm{(2}\omega )$ and ${A}_{\mathrm{3,}x}^{{\rm{F}}}\mathrm{(2}\omega )$ using the Fresnel relations at the boundaries and free-field propagation.

**Figure 5:**
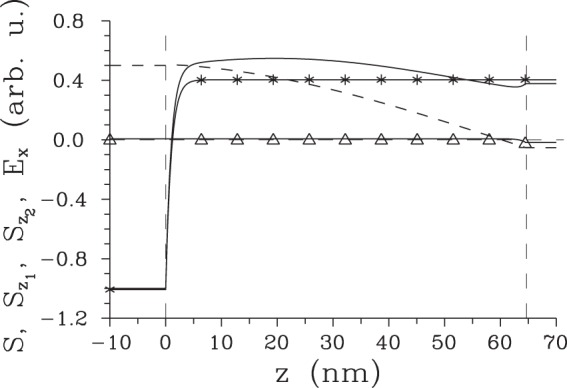
Poynting vector ${S}_{{z}_{1}}$ $[{S}_{{z}_{2}}]$ of the second-harmonic field originating around the boundary at *z*_1_ = 0 [*z*_2_ ≡ *L*] (solid curve with * [Δ]) and the complete Poynting vector *S* (plain solid curve) as functions of position *z* inside a nonlinear layer of thickness *L* = 64.5 nm sandwiched by air and silicon substrate; *n*_2_(*ω*) = 1.5, *n*_2_(2*ω*) = 1.6 and *λ*(*ω*) = 0.8 *μ*m. The second-harmonic electric-field amplitude *E*_*x*_ is plotted as a function of *z* by the decreasing dashed curve. Dashed lines given as *S* = 0, *z* = 0 and *z* = *L* are also drawn.

As an example, using the developed model we numerically analyze the SHG from a centrosymmetric nonlinear layer of thickness *L* = 64.5 nm fabricated on a silicon substrate and surrounded by air (parameters are given in the caption to Fig. 5). The generation of surface second-harmonic field around the boundary at *z*_1_ = 0 is much more intense compared to that around the boundary at *z*_2_ = *L*, as documented by the curves giving the appropriate Poynting vectors ${S}_{{z}_{1}}$ and ${S}_{{z}_{2}}$ in Fig. 5. This unbalance in surface SHG originating around the boundaries at *z*_1_ and *z*_2_ is caused by back-scattering of the second-harmonic field that considerably suppresses the electric-field amplitude *E*_*x*_ in the vicinity of the boundary at *z*_2_ (see the dashed curve in Fig. 5). The complete Poynting vector *S* derived from the second-harmonic field then reflects interference of both terms arising around the boundaries at *z*_1_ and *z*_2_. Comparing the curves for different Poynting vectors drawn in Fig. 5 we reveal that this interference is constructive (destructive) in the area around the boundary at *z*_1_ (*z*_2_). Moreover, the second-harmonic field propagating in air is more than twice intense compared to that propagating in the substrate.

## Conclusions

We have analyzed the process of second-harmonic generation in a finite 1D nonlinear medium using the Green-function technique. Considering identical indices of refraction both for the nonlinear medium and its surroundings, we have suppressed the effects of fields’ back-scattering on the boundaries and isolated this way the substantial (natural) features of surface second-harmonic generation. Assuming the fundamental field propagating along the +*z* direction, we have uniquely identified the terms giving the surface second-harmonic fields generated around both boundaries. We have verified this identification of surface terms by solving the appropriate wave equation by the Fourier-transform method with subsequent inclusion of the boundary conditions for field amplitudes. According to the obtained formulas, the surface second-harmonic fields are generated in a thin layer in the closest vicinity of a boundary. The formula for layer thickness has been derived. The presence of surface second-harmonic fields in the surroundings of the boundaries results in unusual (unexpected) directions of the flow of energy of the second-harmonic field composed of both volume and surface parts: Loosely speaking, the beginning (as well as the end) of the generation of the second-harmonic field in the nonlinear process requires the emission of a small amount of second-harmonic field in the opposed direction to that of the fundamental field because of the electric- and magnetic-field continuity requirements.

We have suggested a simple analytically solvable model for the surface second-harmonic generation in centrosymmetic nonlinear crystals. We have demonstrated the features of surface second-harmonic fields in this model by analyzing two simple and practical examples: one nonlinear boundary and a nonlinear layer placed on a substrate.

## Data Availability

We declare to provide all simulated data used for creation of the figures on request.
